# Complete mitochondrial genome of the hydrothermal vent stalked barnacle *Vulcanolepas fijiensis* (Cirripedia, Scalpelliforms, Eolepadidae)

**DOI:** 10.1080/23802359.2019.1644564

**Published:** 2019-07-22

**Authors:** Won-Kyung Lee, Hyun Mi Kang, Benny K. K. Chan, Se-Jong Ju, Se-Joo Kim

**Affiliations:** a Genome Editing Research Center, Korea Research Institute Bioscience and Biotechnology, Daejeon, Korea;; b Stem Cell Research Center, Korea Research Institute Bioscience and Biotechnology, Daejeon, Korea;; c Biodiversity Research Center, Academica Sinica, Taipei, Taiwan;; d Global Ocean Research Center, Korea Institute of Ocean Science and Technology, Busan, Korea;; e Marine Biology Major, University of Science and Technology, Daejeon, Korea

**Keywords:** *Vulcanolepas fijiensis*, Eolepadidae, hydrothermal vent barnacle, mitochondrial genome, North Fiji Basin

## Abstract

The family Eolepadidae is the only stalked barnacle in hydrothermal vent regions. Here, we determined the mitogenome of the eolepadid *Vulcanolepas fijiensis*. The mitogenome was 17,374 bp long, with 76.6% AT content. Its protein-coding gene organization was identical to that of the deep-sea scalpellid *Arcoscalpellum epeeum*. On the mitogenomic tree, two scalpellomorphan families (Eolepadidae and Scalpellidae) were monophyletic while the other scalpellomorphan family Pollicipedidae did not form the monophyletic group with them. Further mitogenomic analysis of undetermined taxa in hydrothermal vents is required to deepen our understanding of their phylogenetic relationships.

Based on traditional systematics and molecular phylogenetic analysis, hydrothermal vent barnacles are divided into two different ancestor groups, the major vent clade composed of brachylepadomorphan Neobrachylepadidae, verrucomorphan Neoverrucidae and scalpellomorphan Eolepadidae, and the balanomorphan *Eochionelasmus* clade (Herrera et al. 2015). As of 13 June 2019, GenBank contained a single complete mitochondrial genome (mitogenome) of balanomorph *Eochionelasmus ohtai*, but no mitogenomes of the major vent clade. The family Eolepadidae is the only stalked barnacle within the major clade and composes 4 genera including *Ashinkailepas*, *Neolepas*, *Vulcanolepas*, and *Leucolepas* (Herrera et al. 2015). To understand the phylogenetic relationship among hydrothermal vent barnacles, we determined the mitogenome of the eolepadid *Vulcanolepas fijiensis* (Chan et al. 2019).

On 5 December 2016, *V. fijiensis* specimens were collected from North Fiji Basin (16°59′S and 173°55′E; 1988 m depth). Genomic DNA extraction, sequencing, gene annotation, and phylogenetic analyses followed the methods of Kim et al. (2017, 2018). An ambiguous mixed peak Y at nucleotide site 48 of tRNA*^Gln^* was changed to C (forward primer, FBar_F2: TAAGTAGCAACCCTCAAGGT; reverse primer, FBar_R2_N: AGTTCTATGGCTTCGTGGAA) by Sanger sequencing. The specimens have been deposited in the National Institute for Biological Resources, Korea (NIBR-IV0000830370) and the Biodiversity Research Museum of Academia Sinica, Taiwan (ASIZCR-000411).

The complete mitogenome of *V. fijiensis*, which was determined with the same individual obtained the CO1 sequence on GenBank submitted by the original description (Accession no. MH636383; Chan et al. 2019) was 17,374 bp in length (Accession no. MN061491; 76.6% AT content). It is consisted of 13 protein-coding genes (PCGs), two ribosomal RNAs (rRNAs), 22 transfer RNAs (tRNAs), and a non-coding region. The PCG organization was identical to that of the deep-sea scalpellid *Arcoscalpellum epeeum* (Accession no. MH791047). Their tRNAs rearranged between CYTB and ND1 and between 12S rRNA and ND2.

All of the PCGs had an ATN start codon while ND1 was inferred to begin with GTG and COX1 was not determined. Most of the PCGs terminated with a complete stop codon (TAA or TAG), although COX1, ND3, and ND4 had an incomplete stop codon (T––). The 16S and 12S rRNAs were 1316 bp (73.4% AT content) and 730 bp (68.1% AT content), respectively. The tRNA genes ranged from 56 to 70 bp in size. A 890-bp-long (76.6% AT content) non-coding region was located between the 12S rRNA and tRNA*^Trp^*.

Phylogenetic trees were constructed with the PCGs of 18 barnacles using maximum-likelihood (ML) and Bayesian inference (Figure 1). The tree topologies were consistent with those obtained in the previous studies (Herrera et al. 2015; Kim et al. 2018). The eolepadid *V. fijiensis* and the scalpellid *A. epeeum* formed a monophyletic clade with high support values of 90% BP and 1.00 BPP. While the pollicipedid *Pollicipes polymerus* in the intertidal zone did not form the monophyletic group with *V. fijiensis* and *A. epeeum*.

**Figure 1.**
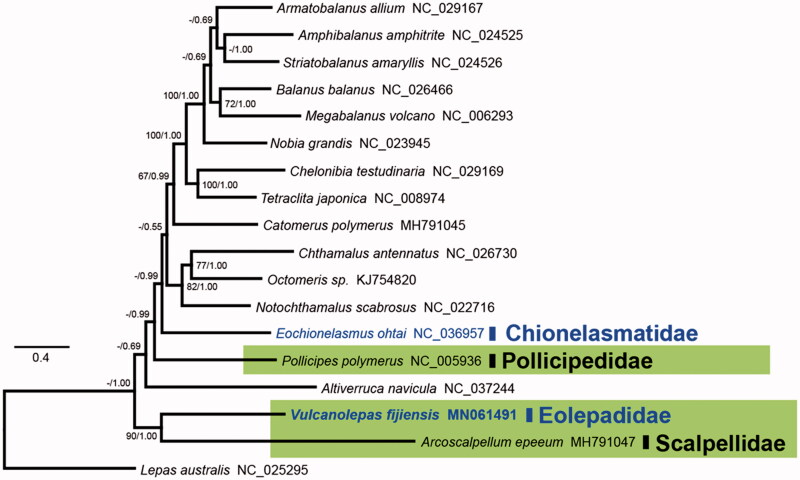
Phylogenetic tree of *Vulcanolepas fijiensis* and other thoracican barnacles based on 13 mitochondrial protein-coding genes. The model GTR + I + G was selected as the best evolutionary model using jModelTest 2.1.4. Green-shaded box contains three families of the order Scalpelliformes. Blue letters indicate the hydrothermal vent barnacle members. Numbers on internodes are the maximum likelihood bootstrap proportions (left) and Bayesian posterior probabilities (right). Bootstrap values >60% are given above the nodes.

Further mitogenomic analysis of undetermined taxa in the major vent clade, Neobrachylepadidae, Neoverrucidae, and other eolepadid genera, is required to deepen our understanding of their phylogenetic relationships.
